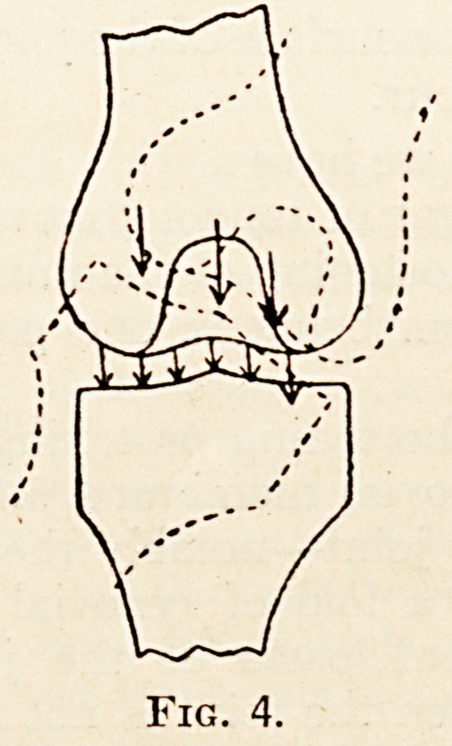# The Operative Treatment of Fractures

**Published:** 1911-01-07

**Authors:** 


### Surgery.

### THE OPERATIVE TREATMENT OF FRACTURES.

### I. GENERAL.

The treatment of a fracture consists in getting the bone to join, and in obtaining a functionally useful limb afterwards. If the bone does not join, the limb is of course useless, but even when there is good firm union the function of the limb may be so impaired that the wage-earning capacity of the individual is very considerably diminished. The Workmen's Compensation Act has in recent years brought this point very prominently into view. The state of the labour market enables employers to pick and choose, and they cannot afford to take a man who has some slight lameness whereby he is rendered more likely to slip or stumble and so to sustain another injury for which the employer may have to pay heavily.

The aim, therefore, of the surgeon who is called upon to treat a case of fracture should be to restore the broken pieces absolutely to that position which they held before. When this is not attained the function of the limb may be impaired in one of the following ways:

(a) *Shortening.*—This, by itself, is not of very great importance, but it is seldom that shortening occurs without one of the other results mentioned below. On the rare occasions when it does occur by itself in the lower limb the loss of function is practically nil; the lessening of wage-earning capacity is, however, very considerable, as the deformity is easily noticed, and to the untrained eye this strongly suggests loss of function.

(b) *Alteration in the Direction of the Lines of Force as they pass from one bony surface to another.*—This result is of extreme importance, as the joint is at a very great disadvantage since it has to work in an abnormal plane. As a result of this the muscles moving the joint have to do more work to produce the same effect; and the results of the increased wear and tear are evidenced by the changes of osteo-arthritis occurring in the joint.

The changes may result from three different forms of mal-union:—

(i) *Lateral displacement* where the distal piece of bone is still in its normal line, but is displaced either outwards or inwards. The impairment from this is not very great, as the plane in which the joint beyond acts is parallel to the normal, but is shifted further out.

(ii) *Angular Displacement.*—In this the bone is bent away from its normal line. Hence not only is the whole joint shifted out, but also the plane of action is deviated through an angle equal to the angle of displacement.

(iii) *Rotation.*—In this the plane of action of the joint below is rotated through the same angle as the bone is rotated. In the first of these three the muscles moving the joint are acting at a disadvantage and must therefore do more work in order to produce the same effect; in the second and third there is the added factor that all the joints distal to the fracture are thrown out of harmony with the joint above, the lines of force pass from one bony surface to another obliquely to the directions that they take in the normal joint.

These points may be best explained by taking fractures of the shaft of the femur as an example. In walking and other important movements of the lower limb the three joints, hip, knee, and ankle, all move in a sagittal plane. In the knee and ankle

Fig. 1. Fig. 2. Fig. 3.

movements are only possible in this plane, in the hip movement in a very large number of planes is possible, but the sagittal plane is the one most frequently used. If, now, the lower end of the femur, with the thigh, be simply displaced outwards (fig. 1), the new plane of action is parallel to the old, and but little harm is done. If, however, angular displacement (fig. 2) has occurred, the knee and ankle are now moving in a plane inclined to the

Fig. 4.

sagittal, so that the patient tends to walk on the inner side of the foot, and the weight is transmitted from the femur to the tibia, and tibia to ankle, in a direction actually vertical, but relative to the normal anatomy of the joint in a direction downwards and inwards (fig. 4).

When the lower fragment and the leg are rotated out (fig. 3) the plane of action is rotated into one approaching the coronal, and in forward progression either the hip has to be strongly rotated in to bring this back into the sagittal plane, or else great strain is put on the inner side of the knee and ankle in the attempt to flex the joints in this abnormal position. These forms of displacement are usually combined.

Considerable stress has been laid on this subject, firstly because of its great importance; secondly because this importance is but little recognised. Again, by a clear understanding of the above factors, an explanation is arrived at of those cases of mal-union with marked distortion of the bone and even 4 inches of shortening, and yet a useful functional limb. In these cases, which, however, are not common, the whole of the displacement is lateral, and so, although the deformity is marked, the plane of action of the joints is parallel with the normal, and so the use of the limb, as a whole, is not much affected.

(c) The third way in which the function of the limb may be impaired after a fracture is by limitation of movement of one or more joints.

Under this head, again, there are several causes. In those cases where the fracture actually passes into the joint it is very liable to be complicated by a partial dislocation unless the broken pieces are returned into their exact position. In this way the relation of the bony surfaces to each other is altered. The impairment of movement resulting from this is increased by the callus that runs across the joint surface along the line of fracture. Since the amount of callus formed is inversely proportional to the exactness of apposition attained, this cause of impairment of movement is also lessened by returning the broken pieces to their exact original position. But very marked limitation of movement may result even when the line of fracture does not pass into the joint. This may be due to mechanical limitation from a mass of callus near the joint coming in contact with the bone on the other side of the joint; or, again, it may be due to "adhesions." The word "adhesions" has long been used to include many different factors. Actual bands of fibrous tissue, the results of inflammatory changes, running from one surface of the joint to the other, do undoubtedly occur.

Besides these we have:

(i) Involvement of tendons moving the joint;

(ii) Actual shortening of the muscles, or a loss of extensibility from being in one position for a long period of time;

(iii) Actual shortening or taking in of the slack part of the synovial membrane and capsule of the joint. In every joint—notably the shoulder-joint—there is always a fold of synovial membrane lying loose in different parts of the joint in different positions. When the joint is not moved for a long period this slack fold disappears, and so when attempts are now made at movement they are ineffectual.

This last is possibly the most frequent, and probably the most important of the various causes usually grouped under the word "adhesions."

It will be noticed that all these occur as a result of the joint being kept in one position for an extended period of time. We may add that the liability of a joint to become fixed as a result of not being moved is directly proportional to the age of the individual. A child's joint may be kept in a plaster splint for months on end without any impairment of movement resulting; in an old person fixation for even a week or less will so impair movement that it is only by great care and with much pain to the patient that the function is restored.

A study of carefully collected statistics has during many years been convincing the profession that the results of the treatment of fractures not only has fallen far short of the ideal but also of the practical possibilities. The *x*-rays have confirmed this view, and the Workmen's Compensation and allied Acts have impressed it on the minds of surgeons. As a result, much work has been done, and two new schools have sprung up in the attempts to improve the after-results of fractures.

The first of these schools—the followers of M. Lucas Championnière—has been content with a moderate reduction of the deformity, without attempting to obtain exact apposition, but has tried to reduce to a minimum the disadvantages dependent on the factors other than the actual displacement. By massage and by early passive movement the excess of callus is limited, and the impairment of movement in the joints is done away with, while the broken bones are kept in as good a position as is possible by means of sandbags and pillows without any rigid retentive apparatus.

While admitting that this school has done much valuable work, it must be pointed out that the ideal aimed at is not so high as is that of the second school, which tries to return the broken ends of bone into that position which they held before they were broken. Of this school there are two branches: there are those who try to attain this end by an improvement of the old methods. This is the school of Bardenheuer, where the broken limb is put up in some retentive apparatus and is then examined by the *x*-rays, and any remaining deformity is corrected by bands and pulleys passing round the limb and dragging now in one direction now in another, with an extension on the limb if necessary of any force up to 100 pounds.

The other branch includes those who cut down upon a simple fracture, replace the broken ends into exactly that position which they had before, and then fix them there by some internal splint, such as a steel plate with screws. This is the school whose ablest exponent and advocate during the last seventeen years has been Mr. Arbuthnot Lane. This is the only method that realises the ideal of exact apposition mentioned above, and is the only one that takes into account all the factors mentioned above as impairing the function of the limb.

By this exact apposition all shortening is done away with, all displacement—lateral, angular, and rotary—is corrected, the normal relation of joint surfaces one to another is kept, and callus is reduced to a minimum. To prevent limitation of movement resulting from one of those caused under the head "adhesions," the methods of the school of Lucas Championnière are brought in as adjuncts to the operative treatment.

(*To be continued.*)

## Figures and Tables

**Fig. 1. f1:**
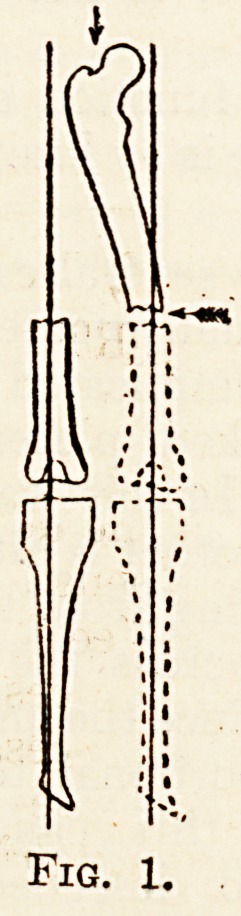


**Fig. 2. f2:**
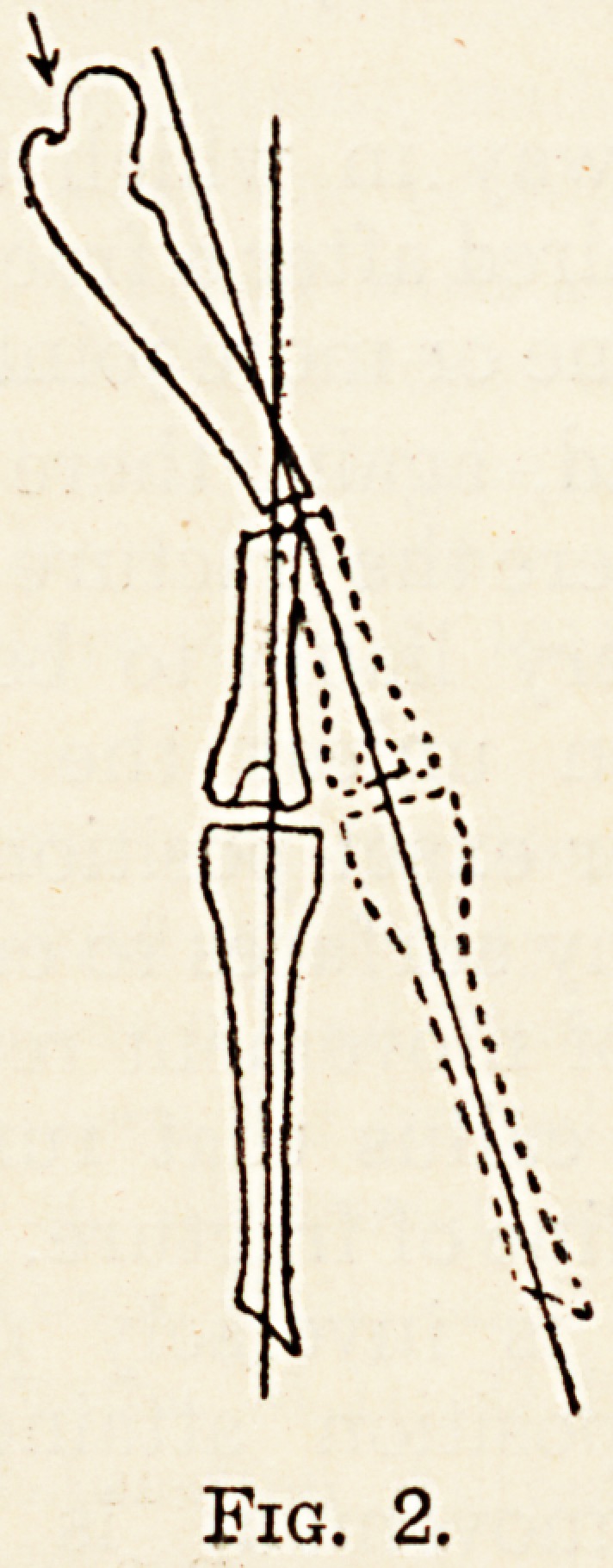


**Fig. 3. f3:**
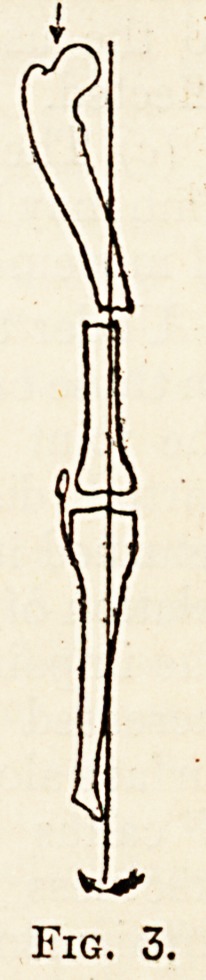


**Fig. 4. f4:**